# Skin Puckering in a Tibial Fracture

**DOI:** 10.7759/cureus.7791

**Published:** 2020-04-23

**Authors:** Patrick McCabe, Kunal Mohan, Prasad Ellanti, Niall Hogan

**Affiliations:** 1 Trauma and Orthopaedic Surgery, St James's Hospital, Dublin, IRL; 2 Trauma and Orthopaedic Surgery, St. James's Hospital, Dublin, IRL

**Keywords:** tibia shaft fracture, dimple, pucker

## Abstract

Skin puckering is a feature observed in fractures that undergo large displacements at the time of initial injury and occur as a result of adherence of the dermal tissues to the underlying fracture fragment. Herein, we discuss the interesting case of a 47-year-old male who suffered a comminuted tibial shaft fracture which resulted in marked pretibial skin puckering prior to fracture reduction with striking corresponding images noted on computerised tomography (CT) scanning.

## Introduction

Skin puckering is a phenomenon associated with cases where significant fracture displacement occurs. It is often associated with fractures of the upper limb, particularly in high-energy proximal humeral fractures and paediatric supracondylar fractures, where its presence is a direct correlation with significant underlying soft tissue trauma [1-3]. It is much less frequently seen in cases of lower limb trauma and, in particular, tibial shaft fractures.

## Case presentation

We describe the intriguing case of a year 47-old-man who suffered a comminuted, spiral midshaft tibial fracture following an assault while on an evening of socialising. The recollection of the event was impeded secondary to a significant volume of alcohol consumed. His past medical history was significant for a conservatively treated ipsilateral Achilles tendon injury, and he was a smoker of a minimum of 20 cigarettes per day.

**Figure 1:**
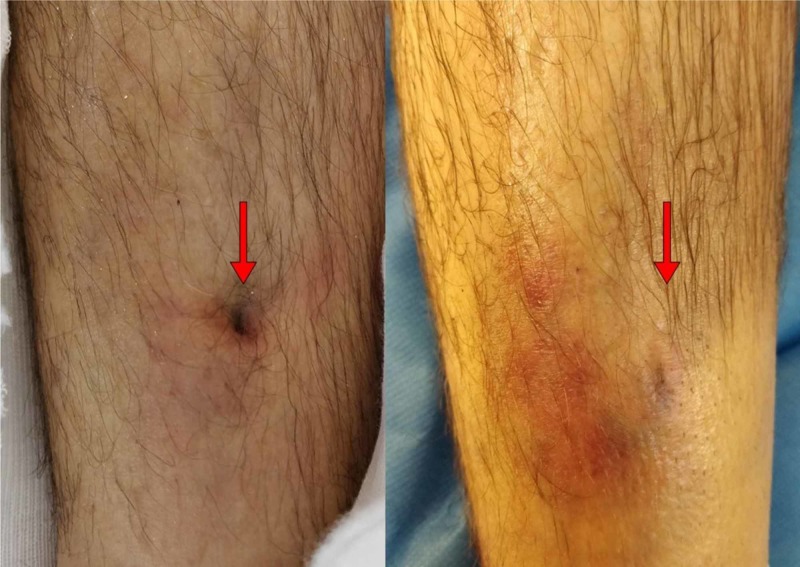
Pre and post-reduction images of the patients right tibia demonstrating “pretibial puckering” of the soft tissues on the left with resolution post-reduction evident on the right

**Figure 2:**
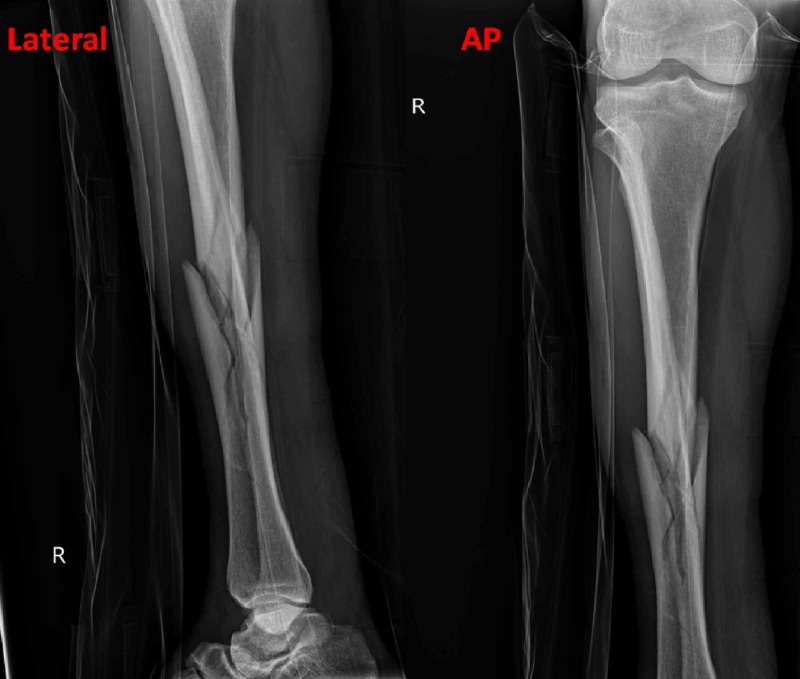
Lateral and anteroposterior (AP) x-ray views of tibia and fibula fracture on presentation

**Figure 3:**
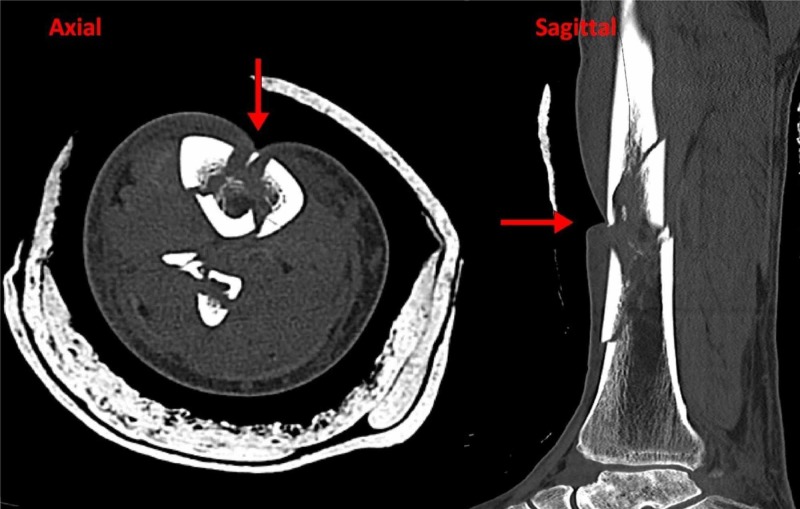
Axial and sagittal computed tomography (CT) images demonstrating tethering of the soft tissues within the fracture site prior to reduction

On presentation to the Emergency Department, it was noted that there was gross deformity present in the patient's right lower limb. The injury was isolated, closed, and there was no discernible neurological or vascular deficit distally. However, there was a pronounced “dimple” or “skin pucker” over the patient’s pretibial area with a surrounding subcutaneous hematoma (Figure 1). Initial x-rays performed demonstrated a comminuted midshaft tibia and fibula fracture but nothing else of significant concern (Figure 2). However, a subsequent computerised tomography (CT) scan highlighted a complex fracture with the invagination of the subcutaneous soft tissues within the fracture site (Figure 3).

Immediately before the definitive operative fixation, an attempted closed reduction of the fracture under anaesthesia was performed, combining both traction and rotation, at which time it was noted that the skin pucker resolved (Figure 1). Fluoroscopic screening at that time demonstrated a satisfactory reduction of the fracture.

The patient underwent definitive fixation with an intramedullary tibial nail and proceeded through a routine postoperative course. Partially protected weight-bearing was commenced immediately, transitioning to full unaided mobilisation by six weeks following the index procedure. Complete fracture union was noted at six months.

## Discussion

It is noteworthy that numerous cases of skin puckering directly correlating to fracture severity are described in relation to the upper limb [1-5]. Alshryda et al. described puckering in the context of proximal humeral fractures [1]. Jindal et al. noted that its presence in the surgical neck of humerus fractures pointed toward a more complicated fracture pattern warranting open, rather than closed, reduction [2]. There are also infrequent reports of its presence in complex high fractures of the distal radius [3]. Skin tethering has been long recognised in the paediatric setting as a predictor of severity in supracondylar humerus fractures, whereby the proximal bony fragment transects the brachialis muscle, ‘puckering’ the deep dermis, thus indicating a significant soft-tissue insult as a direct consequence of fracture displacement. In a case series by Nomura et al., there was a preponderance of grossly displaced fractures and associated neurovascular injury in patients with a pucker sign [4]. It was also noted by Ho et al. that the assessment of soft tissue injury was as important as the radiographic appearance when examining patients with these injury patterns, as exemplified in the example that we present here [5].

Despite this fact, with adequate management, Smuin and Hennrikus noted no significant clinical difference in patient outcomes following prompt appropriate management [6].

While there is ample literature detailing these injuries in the upper limb, there is limited documentation of examples in closed tibial fractures.

## Conclusions

To the best of our knowledge, there are limited case reports with clinical photographs and radiological images detailing the skin puckering in a tibial shaft fracture to this extent. In this case, we demonstrate how the presence of this pucker was directly correlated with a complex fracture pattern and a significant underlying bony and soft tissue injury. However, in consonance with evidence from upper limb fractures with similar initial presentations, by receiving expedited and adequate treatment, the patient had an extremely positive outcome and made a full and timely recovery.
